# Covenants Relating to Hospitals

**Published:** 1910-08-27

**Authors:** 


### COVENANTS RELATING TO HOSPITALS.

In the course of negotiations for the sale of land it sometimes becomes necessary for the vendor to impose terms and conditions which will prevent a hospital being built on his land. A restrictive covenant has to be inserted in the conveyance in order to secure the observance of such a condition. Nice questions arise as to the meaning of this kind of restrictive covenant. It has been decided that under a covenant not to carry on any "trade or business" the carrying on of a pay hospital is a "business," even though not carried on for pecuniary profit. Profit is not the test, but the use of the premises for the business of nursing.

In another case, where a covenant prohibited certain specified "trades or businesses," and added "or any offensive trade," a private lunatic asylum was held not to be included in the covenant; but where it was provided that the lessee should not do anything amounting to "the annoyance, nuisance, grievance, or damage of the lessor or the inhabitants of the neighbouring or adjoining houses," it was held that a special hospital intended for poor outpatients suffering from diseases of the throat, nose, ear, skin, eye, and other diseases was prohibited by the covenant.

Where a covenant had the words "or any offensive or noisy trade, business, or profession whatsoever," but no general restriction as to nuisance or annoyance, it was held in an Irish case that a private hospital or nursing home for surgical or medical (not infectious) cases was offensive within the meaning of the covenant.

